# Hemolytic uremic syndrome caused by Shiga toxin–producing *Escherichia coli* in children: incidence, risk factors, and clinical outcome

**DOI:** 10.1007/s00467-020-04560-0

**Published:** 2020-04-22

**Authors:** Elisa Ylinen, Saara Salmenlinna, Jani Halkilahti, Timo Jahnukainen, Linda Korhonen, Tiia Virkkala, Ruska Rimhanen-Finne, Matti Nuutinen, Janne Kataja, Pekka Arikoski, Laura Linkosalo, Xiangning Bai, Andreas Matussek, Hannu Jalanko, Harri Saxén

**Affiliations:** 1 Department of Pediatric Nephrology and Transplantation, New Children’s Hospital, University of Helsinki and Helsinki University Hospital|, P.O. Box 347, 00029 HUS, Helsinki, Finland; 2 Department of Health Security, Finnish Institute for Health and Welfare (THL), Helsinki, Finland; 3 Department of Children and Adolescents, Oulu University Hospital, Oulu, Finland; 4 PEDEGO Research Unit, Research Unit for Pediatrics, Dermatology, Clinical Genetics, Obstetrics and Gynecology, Medical Research Center Oulu (MRC Oulu), Oulu, Finland; 5 Department of Pediatrics and Adolescent Medicine, Turku University Hospital, Turku, Finland; 6 Department of Pediatrics, Kuopio University Hospital and University of Eastern Finland, Kuopio, Finland; 7 Department of Pediatrics, Tampere University Hospital, Tampere, Finland; 8 Division of Clinical Microbiology, Department of Laboratory Medicine, Karolinska University Laboratory, Stockholm, Sweden; 9 Division of Laboratory Medicine, Oslo University Hospital, Oslo, Norway

**Keywords:** Hemolytic uremic syndrome, Shiga toxin–producing *E.coli* (STEC), Kidney failure, Shiga toxins, Shiga toxin subtypes

## Abstract

**Background:**

Hemolytic uremic syndrome (HUS) is a multisystemic disease. In a nationwide study, we characterized the incidence, clinical course, and prognosis of HUS caused by Shiga toxin (Stx)–producing *Escherichia coli* (STEC) strains with emphasis on risk factors, disease severity, and long-term outcome.

**Methods:**

The data on pediatric HUS patients from 2000 to 2016 were collected from the medical records. STEC isolates from fecal cultures of HUS and non-HUS patients were collected from the same time period and characterized by whole genome sequencing analysis.

**Results:**

Fifty-eight out of 262 culture-positive cases developed verified (*n* = 58, 22%) STEC-HUS. Another 29 cases had probable STEC-HUS, the annual incidence of STEC-HUS being 0.5 per 100,000 children. Eleven different serogroups were detected, O157 being the most common (*n* = 37, 66%). Age under 3 years (OR 2.4), *stx2* (OR 9.7), and *stx2a* (OR 16.6) were found to be risk factors for HUS*.* Fifty-five patients (63%) needed dialysis. Twenty-nine patients (33%) developed major neurological symptoms. Complete renal recovery was observed in 57 patients after a median 4.0 years of follow-up. Age under 3 years, leukocyte count over 20 × 10^9^/L, and need for dialysis were predictive factors for poor renal outcome.

**Conclusions:**

Age under 3 years, *stx2*, and *stx2a* were risk factors for HUS in STEC-positive children. However, serogroup or *stx* types did not predict the renal outcome or major CNS symptoms.

## Introduction

Hemolytic uremic syndrome (HUS) is a complex, multisystemic disease characterized by microangiopathic hemolytic anemia, thrombocytopenia, and impaired kidney function, and it is one of the most common causes of acute kidney injury in children. It is most often caused, especially among children, by Shiga toxin (Stx)–producing *Escherichia coli* (STEC). The serotype O157:H7 has been previously recognized as the predominant cause of HUS worldwide [1]. However, this may arise from the diagnostic bias for this serotype. Other serogroups such as O26, O55, O80, O103, O104, O111, and O145 have been increasingly discovered to cause STEC-HUS as well [2–6]. Shiga toxins (Stxs), encoded by *stx* genes, are the major virulence factors to cause thrombotic microangiopathy, which forms the histopathological basis of HUS. Stxs can be divided into two major types (Stx1 and Stx2) and several subtypes, with Stx2 usually leading to more severe symptoms [7, 8]. Stx subtypes have also been found to differ in toxin potency [8–10]. HUS can sometimes be caused by other infectious agents such as *Streptococcus pneumoniae* and genetic disorders regulating the alternative pathway of the complement system (atypical HUS) [11–13].

STEC is usually contracted through contaminated food or drink, such as undercooked meat and unpasteurized milk or vegetables, or transmitted from person to person. Cases appear both sporadically and in epidemics. The development of HUS among people infected with STEC has varied from 5 to 15% in different studies and has been even higher in some studies [14–17].

The treatment of STEC-HUS is symptomatic with no curative treatment. The use of antibiotics in the treatment has long been considered contraindicated as there has been some evidence that antibiotic use increases the likelihood of developing or worsening HUS [18, 19].

The prognosis of children with HUS has improved over time, mostly due to advances in intensive care and dialysis. Nevertheless, HUS in children is still associated with significant morbidity and mortality. In the acute phase, approximately half of the patients need kidney replacement therapy, and in about 25% of the cases, the disease is associated with neurological symptoms [13, 20]. Mortality varies between 1 and 5% [13, 15, 20–22]. Patients need long-term follow-up since approximately 20–25% develop some degree of chronic kidney disease (CKD) [21, 23]. In addition, 5% develop severe sequelae such as kidney failure or neurological damage [13, 21]. The length of oligo-anuria or dialysis are found to be the most significant risk factors of poor kidney prognosis, with the occurrence of neurological symptoms, proteinuria, hypertension, dehydration, high leukocyte, or hematocrit levels appearing to have some prognostic value as well [21, 23, 24].

The purpose of this study was to estimate the annual incidence of HUS among children < 17 years of age in Finland and to characterize clinical features, treatment, and prognosis of patients with STEC-HUS in a nationwide study. We also aimed to investigate whether microbiological factors affect the risk of developing HUS and the severity of the disease and long-term outcome.

## Methods

### Collection of patient data

Patient data collection was performed retrospectively using the hospital records and the National Infectious Diseases Register. In 2000–2016, the five University Hospitals reported STEC findings from a total of 262 pediatric patients (< 17 years of age) to the National Infectious Diseases Register, as required by the Finnish legislation. STEC isolates from fecal samples of pediatric patients were sent to the Finnish Institute for Health and Welfare (THL) for further analysis. Isolates from all pediatric patients (*n* = 262) with or without HUS were included in this study. The fecal samples were collected because of clinical suspicion of HUS and differential diagnostics of diarrhea or due to screening of STEC in HUS patient contacts.

In addition to the STEC-positive patients gathered from the National Infectious Diseases Register, we retrospectively identified all pediatric patients diagnosed and treated for HUS in the five University Hospitals using the corresponding ICD-10 diagnosis code D59.3. In addition, all patients with the diagnosis code N17.9 for unspecified acute kidney injury were reviewed, and cases that filled the diagnostic criteria for HUS were included.

The clinical criteria for HUS were hemolytic anemia with a hemoglobin (Hb) level < 10 g/dL, thrombocytopenia with a platelet count < 150 × 10^9^/L, and acutely reduced kidney function with a plasma creatinine concentration above the upper normal limit for age. None of the patients included in the study showed clinical or laboratory signs of other possible disorders (SLE, glomerulonephritis) in the follow-up by pediatric nephrologists. A verified STEC-HUS case was defined as a patient with clinical HUS and a culture-positive stool sample. A probable STEC-HUS case was defined as a patient with clinical HUS and the evidence of *stx* gene (by polymerase chain reaction) or Stx toxins (by immunological methods) in an enriched stool culture. In addition, culture- or Stx toxin–negative patients with a history of diarrhea prior to the development of HUS and HUS cases with at least one family member positive for STEC in the stools were regarded as probable cases.

For the calculation of the overall incidence of HUS in the Finnish pediatric population, we also collected data on HUS cases caused by other infectious agents, such as *Streptococcus pneumoniae,* or cases with atypical HUS. These 13 patients were not included in the further analysis. The size of pediatric population during the study period was obtained from Statistics Finland (https://www.stat.fi).

The clinical and laboratory data of patients were collected from the medical records until the most recent follow-up visit. The data included patient’s gender and age at the time of hospitalization, symptoms, clinical findings, treatment, e.g. need, type, and length of dialysis or plasmapheresis, use of antimicrobial or other medication, and also the possible administration of red blood cells or platelets. The usual treatment policy was to preserve the Hb above 6.0–7.0 g/dL. Platelet transfusions were performed only in the case of bleeding or prophylactically before surgical procedures, such as placement of dialysis catheters. Laboratory data on Hb, leukocyte count, platelet, blood urea nitrogen, creatinine, lactate dehydrogenase, cystatin C, transaminase, and amylase levels were collected. For assessing the long-term outcome, glomerular filtration rate (GFR) was evaluated using either ^51^Cr-EDTA measurement or estimated (eGFR) from the CKiD Schwartz formula [25]. Kidney function was considered decreased at the last follow-up if GFR was under 90 mL/min/1.73m^2^ [26]. The limit for proteinuria was protein-to-creatinine ratio > 20 g/mol or daily urine protein excretion (dU-Prot) > 200 mg. Hypertension was defined as systolic or diastolic pressure ≥ 95^th^ percentile for sex, age, and height [27]. Long-term outcome was reported only for patients with a follow-up of at least 1 year. The national multicenter approach of this study allowed reliable collection of follow-up data of the patients. Long-term renal outcome was classified as poor if the patient had decreased GFR, proteinuria, hypertension, or medication for the latter two.

### Microbiological characterization by whole genome sequencing

Whole genome sequencing (WGS) for available isolates (*N* = 262) was performed by using MiSeq or HiSeq sequencers (Illumina) as part of different projects at THL (*N* = 58), at Institute National de Saude Doutor Ricardo Jorge (INSA), Lisbon, Portugal (*N* = 44), and at SciLifeLab, Stockholm, Sweden (*N* = 160). Bacterial DNA extraction and genomic library preparation using Nextera chemistry (Illumina) were performed by using methods and procedures in each laboratory as previously described [28, 29].

The WGS data analysis was performed at Karolinska Institutet, Sweden. The sequencing reads were quality-control processed and quality evaluated with QCtool pipeline (https://github.com/mtruglio/QCtool). The processed reads were assembled de novo with SPAdes (version: 3.12.0) in “careful mode” [30]. The assemblies were annotated with Prokka (version 1.11) [31].

### Determination of serotypes, stx types, and subtypes and *eae*

The assemblies were compared with VirulenceFinder database (DTU, Denmark) (https://bitbucket.org/account/user/genomicepidemiology/projects/DB) using BLAST+ v2.2.30 [32] to determine *stx* genotypes and the presence of intimin-encoding gene *eae*. The cut-off values for gene identity and alignment coverage were set to 90% in the VirulenceFinder database search. Serotype was determined by comparing assembly sequences to the SerotypeFinder database using BLAST+ v2.2.30. In six cases, the samples were not available at the time of study, and information concerning serotype, *stx*, and *eae* was missed.

### Statistical analysis

Statistical analyses were performed with SPSS (version 25) for Windows (IBM Corp, Armonk, NY). Normally distributed variables are reported as means and standard deviations (SD) and comparison between the groups analyzed with Student’s *t* test. Variables that did not show normal distribution are reported as medians and their interquartile ranges (IQR) and analyzed with Mann-Whitney U test. The chi-square test was used to compare distributions of categorical variables between groups. Risk factors for HUS, major central nervous system (CNS) symptoms, and renal outcome were assessed using a binary logistic regression model (for HUS, also adjusting for age and sex) and for long-term renal outcome, also using multivariate analysis. In these analyses, age groups were < 3 years and ≥ 3 years. The results of logistic regression analyses are reported as odds ratios with 95% confidence intervals and two-tailed *p* values. Statistical significance was defined as *p* < 0.05.

### Ethics

The Ethics Committee of the University of Helsinki approved the use of patients’ information and the study protocol.

## Results

### Incidence of STEC infection and HUS

Fifty-eight out of the 262 (22%) children with STEC isolated from the stool had verified STEC-HUS (Table 1). Of these, 56 isolates were available for serotyping and determination of *stx* genotype by WGS. Twenty-nine cases had probable STEC-HUS, including those with both culture- and toxin-negative diarrhea and clinical HUS (20/29), toxin-positive but culture-negative clinical HUS (3/29), and clinical HUS cases with a STEC positive family member (6/29). Thus, altogether 87 cases with either verified (58, 67%) or probable (29, 33%) STEC-HUS were recognized, the total number of patients being 291 (Table 1). No recurrent cases of HUS were seen. Verified and probable STEC-HUS cases did not differ by age, gender, duration of symptoms before hospitalization, or frequency of diarrhea or bloody diarrhea.

**Table 1:** The study population and data sources

Non-HUS STEC cases, all	204
STEC-HUS cases, all	87
Verified STEC-HUS cases^1^	58
Probable STEC-HUS cases, all	29
Culture negative, PCR positive^2^	3
Bloody diarrhea, culture and PCR negative^2^	20
Clinical HUS, family member+^2^	6
Total	291
Non-STEC HUS, all^2^	13
*Streptococcus pneumonia*	5
*Streptococcus pneumoniae* and Influenza A	1
*Campylobacter*	1
Complement defect	4
*Escherichia coli* urinary tract infection	2

^1^From the National Infectious Diseases Register

^2^From patient records

Among Finnish children, the average annual incidence of confirmed and probable STEC-HUS cases was 0.50 per 100,000 and that of all verified STEC cases was 1.5 per 100,000. During the same study period, 13 children had HUS caused by non-*E. coli* etiology. Seven of these were associated with infections caused by *Streptococcus pneumoniae* (*n* = 5), *Streptococcus pneumoniae*, and Influenza A (*n* = 1) or *Campylobacter* (*n* = 1). Two cases had urinary tract infection caused by *E. coli.* Four patients had atypical HUS including complement factor H mutation (*n* = 2), complement factor I mutation (*n* = 1), and membrane cofactor protein mutation (*n* = 1). Thus, the overall annual incidence of HUS was 0.57 per 100,000 children.

**Fig. 1:**
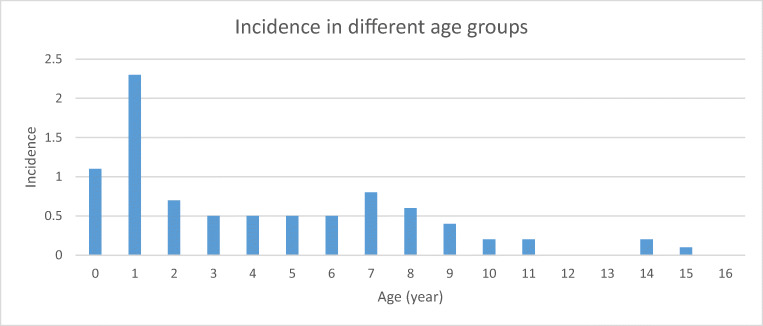
Incidence of STEC-HUS according to age groups of pediatric patients, Finland 2000–2016 (*n* = 87)

Fifty-five of 87 (63%) cases with confirmed or probable STEC-HUS were female, the risk of developing HUS being higher among females than males (OR 2.01, CI 1.20–3.37, *p* < 0.01). The median age of patients with HUS was 3.3 years (1.4–7.5). The incidence was highest in the age group of 0- and 1-year-old children (Fig. 1), < 3 years of age being a risk factor for STEC-HUS (OR 2.36, CI 1.40–3.99, *p* < 0.005).

### Microbiological characteristics

**Fig. 2:**
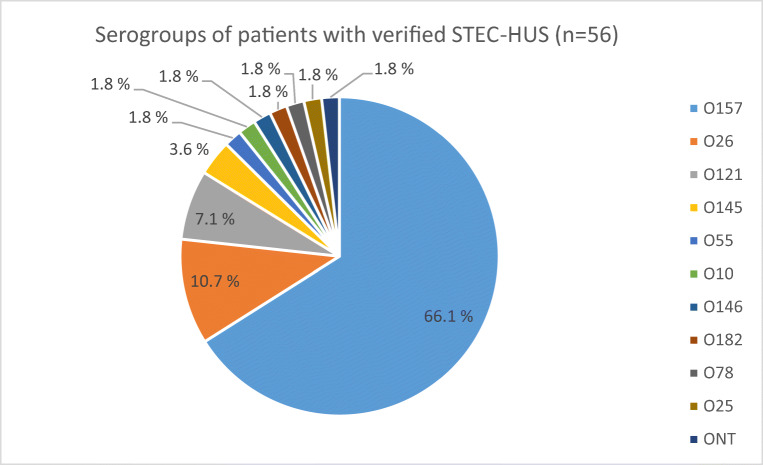
Serogroups of STEC isolates in pediatric patients with HUS, Finland 2000–2016. ONT O non-typeable

O157 was the most frequent (37/56, 66%) serogroup in cases with STEC-HUS, followed by O26 serogroup (6/56, 11%). Altogether 11 different serogroups were found to cause STEC-HUS (Fig. 2). Two isolates were positive for *stx1* only (1 *stx1a* and 1 *stx1c*), 51 for *stx2* only (49 *stx2a,* 1 *stx2b*, *and* 1 *stx2c*), and 3 for both *stx1* and *sxt2* (all *stx1a* and *stx2a*). Cases with O157 and non-O157 STEC-HUS did not differ with regard to presence of *stx1* or *stx2*. Most of the cases (88%) had *eae*-positive STEC. Among patients with STEC infection, the *eae* gene was significantly associated with the serogroup O157 when compared with non-O157 cases (98% vs. 75%, *p* < 0.001).

Among the STEC-positive cases, serogroup O157 was slightly more likely to cause HUS as compared with non-O157 serogroups but without significant difference (OR 1.83, *p* = 0.055) (Table 2). After adjustment for sex and age, the difference was, however, significant. *Stx2,* and especially *stx2a* subtype were found to be risk factors for HUS (*p* < 0.001) (Table 2), while *stx1* alone or together with *stx2* seemed to lower the risk of developing HUS (Table 2). Although *eae* was found in 93% of cases with HUS, the risk of developing HUS was not found to be significantly increased among patients with *eae* (Table 2).

**Table 2:** Serogroups and toxins as risk factors for HUS in 256 STEC positive patients using binary logistic regression model

	HUS (56)	STEC without HUS (200)	OR (95% CI)	*p* value	Adjusted OR*	*p* value
Serogroup O157	37 (66%)	103 (52%)	1.83 (0.99–3.41)	NS	2.14 (1.11–4.12)	0.02
Serogroup O26	6 (11%)	20 (10%)	1.08 (0.41–2.83)	NS	1.12. (0.41–3.19)	NS
Serogroup O121	4 (7%)	4 (2%)	3.77 (0.91–15.58)	NS	2.66 (0.61–11.60)	NS
Stx1(with or without Stx2)	5 (9%)	118 (59%)	0.07(0.03–0.19)	< 0.001	0.07 (0.03–0.18)	< 0.001
Stx1 alone	2 (4%)	54 (27%)	0.10 (0.02–0.43)	0.002	0.09 (0.02–0.39	0.001
Stx1a	4 (7%)	108 (54%)	0.07 (0.03–0.19)	< 0.001	0.07 (0.02–0.19)	< 0.001
Stx2 (with or without Stx1)	54 (96%)	147 (74%)	9.74 (2.29–41.33)	0.002	11.03 (2.56–47.53)	0.001
Stx2 alone	51 (91%)	82 (41%)	14.68 (5.62–38.36)	< 0.001	14.84 (5.62–39.20)	< 0.001
Stx2a	51 (91%)	76 (38%)	16.64 (6.36–43.54)	< 0.001	17.41 (6.55–46.27)	< 0.001
Stx2c	1 (2%)	56 (28%)	0.05 (0.01–0.35)	0.003	0.47 (0.06–4.00)	NS
Stx1 and Stx2	3 (5%)	65 (33%)	0.12 (0.04–0.39)	< 0.001	0.12 (0.04–0.42)	0.001
*eae*	52 (93%)	172 (86%)	2.12 (0.71–6.31)	NS	1.92 (0.63–5.81)	NS

*CI* confidence interval, *NS* nonspecific, *OR* odds ratio, *aOR* adjusted odds ratio with confidence intervals for age groups < 3 and ≥ 3 years and sex

### Clinical features of STEC-HUS cases

The median duration of symptoms before the patient was admitted to hospital was 5 (3–7) days. Diarrhea preceded HUS in 80 (92%) cases, being bloody in 49 (61%). Vomiting was the second most common symptom (67%), followed by fever (37%) and abdominal pain (29%). Hb declined to the median nadir value of 6.0 (5.6–6.6) g/dL and platelets to 31 (22–44) × 10^9^/L. The median creatinine level at the time of hospitalization was 106 (50–303) μmol/L and peak value 343 (117–480) μmol/L. Forty-four (51%) patients progressed to anuria. Twenty-nine patients (33%) developed major CNS symptoms. The renal, CNS, and extrarenal findings are presented in more detail in Table 3.

**Table 3:** Clinical consequences of 87 patients with STEC-HUS

Variables	*n* (%)
Anuria	44 (51)
Oliguria	25 (29)
Major CNS symptoms	29 (33)
Seizures	24 (28)
Impaired consciousness	15 (17)
Hemiparesis	4 (5)
Minor CNS symptoms	12 (14)
Lethargy	8 (9)
Irritability	2 (2)
Vision abnormality	1 (1)
Speech abnormality	1 (1)
Fluctuating hemiparesis	1 (1)
Hypertension	30 (34)
Extrarenal manifestations	
Elevated transaminase level	43 (49)
Pleural effusion	17 (20)
Elevated amylase level	13 (15)
Pericardial effusion	6 (7)
Ascites	5 (6)
Decreased myocardial function	4 (5)
Pulmonary embolism	1 (1)
Rhabdomyolysis	1 (1)
Gallstones*	1 (1)

*Developed 1 month after the acute episode

### Treatment

Fifty-five patients (55/87, 63%) needed dialysis for a median of 10 (6–16) days. Twenty-five of the 55 patients (45%) required dialysis for more than 10 days. Treatment modalities were hemodialysis (HD) in 47 cases, peritoneal dialysis (PD) in six cases, and both HD and PD in two. The number of dialysis sessions varied from one to 41. Two patients became dependent on dialysis and were subsequently transplanted (8 months and 1.5 years after the development of HUS, respectively). Plasma exchanges (PE) were performed on seven patients (before the year 2012). Red blood cell transfusions were given to 74 out of 87 (85%) patients (median three transfusions per patient) to maintain an adequate Hb level. Platelet transfusions were given to 47 out of 87 (54%) (median one transfusion per patient). Forty-seven (54%) patients received antimicrobial treatment. In two cases (4%), the treatment was started prior to the hospitalization, in 9 (19%) before starting dialysis, and in 37 cases (79%) later during the hospitalization. Two patients received eculizumab due to severe neurological symptoms. Median treatment time in the hospital during the acute phase was 17 (12–24) days.

### Outcome

Long-term follow-up data (> 12 months) was available on 77 HUS cases. The median follow-up time was 4.0 (2.0–6.4) years. The overall outcome of children with HUS was good, with no mortality. Eight patients (10%) had mildly decreased kidney function (GFR 60–90 mL/min/1.73 m^2^) and two (2.6%) had more severely affected GFR (31 and 45 mL/min/1.73 m^2^) at the end of follow-up. In addition, two (2.6%) patients had progressed to CKD stage 5 and received a kidney transplantation. Altogether 10 (13%) patients had proteinuria, eight (10%) had persistent hypertension, and nine (12%) had medication for either hypertension and/or for proteinuria (angiotensin-converting enzyme-inhibitor or angiotensin II receptor blocker in eight and beta-blocker in one) at the end of follow-up. Two cases (2.6%) suffered from neurological long-term complications.

### Risk factors of renal and neurological sequelae

Peak leukocyte count over 20 × 10^9^/L and anuria and Hb level over 9.5 g/dL at the time of admission were predictive of dialysis treatment (OR 3.19, *p* < 0.05, OR 121.18, *p* < 0.001, and OR 2.75, *p* < 0.05, respectively) (Table 4). The plasma creatinine (246 vs. 69 μmol/L, *p* < 0.001) and lactate dehydrogenase (2500 vs. 1465 U/L, *p* < 0.005) levels at the time of admission and the peak urea value (35.1 vs. 18.2 mmol/L, *p* < 0.001) were also more likely to be higher among patients needing dialysis. These children had also more often received antimicrobials than patients without dialysis (40/54, 74% vs. 7/32, 22%, *p* < 0.001). If only those patients in whom antimicrobial treatment was started before dialysis were taken into account, the use of antibiotics did not seem to increase the risk of dialysis (Table 4).

**Table 4:** Comparison of clinical and microbiological findings in patients on temporary dialysis (*n* = 55) with those not needing kidney replacement therapy (*n* = 32) as well as in patients with (*n* = 29) and without (*n* = 58) major CNS symptoms using binary logistic regression model

Number	Short-term kidney function			Major CNS symptoms		
	Dialysis (55)	No dialysis (32)	OR (95% CI), *p* value	CNS (29)	No CNS (58)	OR (95% CI), *p* value
Age < 3 years	25 (45%)	16 (52%)	0.83 (0.35–2.00), NS	16 (55%)	25 (43%)	1.63 (0.66–3.99), NS
Antimicrobial treatment, all	40 (74%)^a^	7 (22%)	10.20 (3.62–28.75), < 0.001	22 (76%)	25 (44%)^a^	10.20 (3.62–28.75), < 0.001
Antimicrobial treatment, started before dialysis^c^	9 (17%)^a^	7 (22%)	0.71 (0.24–2.15), NS	3 (10%)	25 (44%)^a^	0.15 (0.04–0.54), 0.004
Leukocytes > 20 E9/L	30 (57%)^b^	9 (29%)^a^	3.19 (1.24–8.22), 0.02	18 (64)^a^	21 (38%)^b^	3.19 (1.24–8.22), 0.02
Hemoglobin > 9.5 g/dL	33 (62%)	12 (38%)	2.75 (1.11–6.80), 0.03	19 (66%)	26 (46%)^b^	2.19 (0.87–5.55), NS
Presence of anuria	43 (80%)^a^	1 (3%)	121.18 (14.86–988.16), < 0.001	20 (69%)	24 (42%)^a^	3.06 (1.19–7.88), 0.02
O157^d^	26 (74%)	11(52%)	2.62 (0.84–8.24), NS	15 (79%)	22 (59%)	2.56 (0.71–9.23), NS
Stx1 alone^d^	2 (6%)	0 (0%)		1 (5%)	1 (3%)	2.00 (0.12–33.86), NS
Stx2 alone^d^	31 (89%)	20 (95%)	0.39 (0.04–3.72), NS	17 (89%)	34 (92%)	0.75 (0.11–4.92), NS
Stx1 and Stx2^d^	2 (6%)	1 (5%)	1.21 (0.10–14.24), NS	1 (5%)	2 (5%)	0.97 (0.08–11.46), NS

*OR* odds ratio, *CI* confidence interval, *NS* nonspecific, *CNS* central nervous system

^a^Data missing from one patient

^b^Data missing from two patients

^c^Data analyzed taking into account only those cases, in whom the antimicrobial treatment was started before dialysis in the dialysis group

^d^Includes 56 patients [(1) renal short-term outcome analysis: 35 with dialysis and 21 without and (2) major CNS symptoms; 19 with CNS symptoms and 37 without], on whom whole genome sequencing data were available

Patients needing dialysis more often had major neurological symptoms than those treated without dialysis (25/55, 45% vs. 4/32, 13%, *p* < 0.005). Patients with major CNS symptoms had also higher creatinine, peak urea, and leukocyte counts than those with either mild or no neurological signs (median peak creatinine 426 vs. 253 μmol/L, *p* < 0.05, median peak urea 34.4 vs. 26.1 mmol/L, *p* < 0.05 and median peak leukocyte count 22.2. vs. 15.5 × 10^9^/L, *p* < 0.005). Peak leukocyte count over 20 × 10^9^/L and anuria predicted major CNS symptoms (Table 4).

Risk factors of poor long-term renal outcome were age under 3 years (OR 5.91), dialysis treatment in general (OR 7.03) and dialysis > 10 days (OR 4.14), and leukocyte count over 20 × 10^9^/L (OR 4.36) (Table 5). In addition, the length of anuria was associated with a worse outcome, the median time of anuria being 9 (0–16) days in patients with renal sequelae at the end of follow-up compared with 0 (0–8) days in patients with good long-term renal outcome. In a multivariate analysis, only age under 3 years and dialysis remained independent risk factors (OR 8.4 and 10.6, *p* < 0.01).

**Table 5:** Comparison of patients with poor renal outcome (decreased GFR, proteinuria, and/or hypertension with or without medication) (*n* = 20) to those with no renal impairment (*n* = 57) using binary logistic regression model

Number	Renal outcome			
	Poor outcome (20)	Good outcome (57)	OR (95% CI)	*p* value
Age < 3 years	16 (80%)	23 (40%)	5.91 (1.75–19.96)	0.004
Antimicrobial treatment	15 (75%)	28 (50%)^a^	3.00 (0.96–9.38)	NS
Leukocytes > 20 × 10^9^/L	15 (75%)	22 (41%)^b^	4.36 (1.38–13.76)	0.012
Platelet infusions	10 (50%)	31 (57%)^b^	0.74 (0.27–2.08)	NS
Major CNS symptoms	10 (50%)	19 (33%)	2.00 (0.71–5.63)	NS
Presence of anuria	14 (70%)	25 (45%)^a^	2.90 (0.97–8.62)	NS
Need of dialysis	18 (90%)	32 (56%)	7.03 (1.49–33.19)	0.014
Dialysis time over 10 days	11 (55%)	13 (23%)	4.14 (1.4–12.14)	0.010
Serogroup O157^c^	8 (57%)	26 (70%)	0.56 (0.16–2.01)	NS
Stx1 alone^c^	1 (7%)	1 (3%)	2.77 (0.16–47.56)	NS
Stx2 alone^c^	12 (82%)	34 (96%)	0.53 (0.08–3.56)	NS
Stx 1 and 2^c^	1 (7%)	2 (5%)	1.35 (0.11–16.13)	NS

*OR* odds ratio, *CI* confidence interval, *CNS* central nervous system, *NS* nonspecific

^a^Data missing from one patient

^b^Data missing from three patients

^c^Includes only 51 patients (14 with poor and 37 with good long-term renal outcome)

Serogroups and *stx* types as a risk factor could be analyzed in 56 HUS patients with WGS information available. Serogroup O157 or *stx* types or were not linked with the need for dialysis (Table 4). Serogroup and *stx* types were not found to be risk factors for major CNS symptoms or for poor long-term renal outcome either (Tables 4 and 5). No difference in the outcome (or dialysis, CNS symptoms, or poor long-term renal outcome) was found based on whether STEC was isolated from the stool or not.

## Discussion

In this nationwide survey, we analyzed the epidemiological, bacteriological, and clinical data of 87 pediatric patients who suffered from HUS caused by STEC during a 17-year period in Finland. The majority (34/56, 61%) of the HUS cases were caused by STEC serogroup O157 and Shiga toxin subtype *stx2a*. As expected, kidneys and CNS were the main target organs and nearly two thirds of the patients needed dialysis for a median of 10 days. Neurological findings were present on admission or developed during hospitalization in one third of our patients. Although the overall outcome of the patients was good, two patients (2.3%) needed kidney transplantation later.

In this study, the incidence of STEC infection (1.5/100,000) was consistent with many earlier reports, although somewhat lower than recently reported in Sweden [33–35]. During the study period, a total of 262 STEC cases were reported in Finland. Of these, 22% progressed to HUS, which is somewhat higher than reported earlier, but equals the frequency in the recent German outbreak [1, 3, 10, 17, 36]. However, the true number of STEC infections is most probably higher as it is likely that stool cultures were not taken from all positive patients and the detection rates are not complete.

The average annual incidence rate of HUS in Finland (0.57 cases per 100,000 children) equals the incidence rates in Germany, Austria, British Isles, France, and Norway [14, 15, 20, 33]. Consistent with most of the previous studies [10, 14, 20, 37], the majority (66%) of HUS cases in Finland were caused by STEC serogroup O157. The proportion of non-O157 serogroups (O26, O80, O103, O104, O145), however, has increased since the early 2010s [10, 33]. A similar change has also been reported from Switzerland and Australia, where O157:H7 serotype is nowadays a minor cause of HUS [13, 38]. Other serotypes, such as O26:H11/H-, have been associated with HUS patients with a potentially severe clinical picture [6]. In our survey, 11% of the cases with STEC-HUS were caused by O26 serogroup strains.

There is controversy as to whether the STEC serotype is a risk factor for developing HUS [8, 10, 17, 35]. In our study, the risk of developing HUS did not differ among patients with serogroup O157 when compared with non-O157 STEC cases or when patients with serogroup O26 were compared with others, but after adjustment for age and sex, serogroup O157 was found to increase the risk of HUS. An association between the development of HUS and the presence of *stx2 gene*, especially subtype *stx2a* (with or without *stx2c*) and *stx2d*, has been described [6, 8, 10, 39, 40]. We found *stx2a* to be a risk factor, whereas *stx1*, *stx1a*, and *stx2c* were associated with a reduced risk of developing HUS. Patients having both *stx1* and *stx2* had lower risk of developing HUS than patients with only *stx2,* consistent with earlier literature [1]. Several other virulence factors contribute to the pathogenicity of STEC, and one of them is *eae*, which encodes intimin [8]. In our material, however, *eae* was not found to significantly increase the risk of developing HUS.

Among the STEC-positive children, age < 3 years was a risk for HUS, which is consistent with the recent findings in Norway [14]. Previously, age less than 5 years has been reported to be a risk for development of HUS [8, 10, 36]. In a recent study, females were more likely to develop HUS [36], as was also the case in our study. STEC-HUS was rare in children over 10 years of age and other etiologies must therefore be kept in mind and ruled out in teenage HUS patients.

The clinical picture of most HUS patients was typical: the majority of the patients had bloody diarrhea but no fever. Half of the patients developed anuria and 63% needed dialysis for a median of 10 days, as seen in earlier studies [20, 33, 41]. Pleural and/or pericardial effusion, ascites, transient elevation in liver enzymes, and/or amylase and hypertension were quite common, observed in almost half of the patients. About half of the patients had received platelet transfusions, in most cases before inserting a hemodialysis catheter. During the early years of this study, platelets were given also before PD-catheter placement, which has recently been shown to be unnecessary [42]. A third of the patients had major neurological symptoms, which is more than reported in previous studies [14, 20]. We do not know the explanation for this. Importantly, however, the outcome of the patients was optimistic with no deaths and neurological sequelae in only two patients. While neurological manifestations are the main cause of acute mortality in HUS (2–5%) [13, 20, 22, 37, 41], none of our patients died. Seven children with severe neurological symptoms were treated with PE, which was used as a treatment modality in many centers, especially in cases with neurological symptoms [11]. After cumulative evidence that PE brought no short- or long-term benefits and could be even harmful [43, 44], it was discontinued in Finland. In our study, however, patients treated with PE did not develop long-term neurological complications, nor did they need dialysis more frequently than others (data not shown). Two of our patients received eculizumab due to severe neurological symptoms. One child had a mild hemiparesis and decreased kidney function (GFR < 60 mL/min/1.73m^2^) at the end of follow-up. The other child recovered fully.

About half of our patients received antimicrobials, usually not directed against STEC or HUS per se. The main indications were suspicion of secondary infections (or prevention of intravenous or urinary catheter or, for example, rising infection parameters and stomach pain causing fear of bowel perforation). The current general opinion is that antibiotics should be avoided at least during the diarrheal phase since they may increase local Shiga toxin concentrations due to the lysis of toxin-containing bacteria [18, 36, 45]. Although a recent meta-analysis [19] failed to show any associations between antibiotic use and higher incidence of HUS, antibiotics are still not recommended in STEC infections or during HUS. In our study, the use of antimicrobial medication was more common in patients who needed dialysis. However, it was usually started after the initiation of dialysis. When only those cases in whom antibiotics were started before the dialysis were taken into account, antibiotic use did not seem to predict the need for dialysis treatment or increase the risk of development of neurological symptoms. No association was seen between antibiotic use and poor long-term outcome.

Children with STEC-HUS are at risk for long-term complications. Complete renal recovery (normal GFR, no proteinuria, and no hypertension) was seen in 57 of 77 (74%) patients after a median follow-up of 4.0 years. This is in line with an earlier meta-analysis showing long-term renal sequelae (hypertension, GFR < 80 mL/min/.173m^2^, and/or proteinuria) in 25% of the patients [23]. In two studies with longer follow-up (9.6 and 8.8. years), the frequency of renal-related sequelae was even higher, 39% and 47%, respectively [24, 46]. In our study, two (2.3%) patients progressed to CKD stage 5 and received a kidney transplant, consistent with the earlier literature [13, 21]. Similarly, two children (2.3%) suffered from long-term neurological complications.

Identification of risk factors for severe disease and poor long-term outcome would help in managing STEC-HUS children. Young age (< 3 years) and dialysis > 10 days were clearly risk factors for later renal sequelae. This is in line with the study of Oakes et al. [46], who found that anuria over 10 days substantially increased the risk for low GFR and proteinuria. Creatinine, leukocytes, Hb, lactate dehydrogenase, and urea were significantly higher among patients needing dialysis. Blood leukocyte count > 20 × 10^9^/L during the acute phase and Hb > 9.5 g/dL on admission also predicted the need for later dialysis. Interestingly, leukocytes > 20 × 10^9^/L also predicted major CNS symptoms. High leukocyte count and hemoconcentration have both been found to be associated with higher risk of death [22]. Twenty-five (86%) of the 29 cases with major CNS symptoms needed dialysis. In 20 children, the CNS symptoms started before dialysis (median 2 days after the hospitalization), which speaks against an association between dialysis and neurological manifestations.

According to our results, serotype, *stx1/stx2* type, and presence of the *eae* gene could not predict which HUS patients were at risk of developing more severe clinical outcome (e.g. need of dialysis, CNS symptoms) or long-term renal sequelae. In one earlier study, serotype O157:H7 was associated with a longer need for dialysis treatment [20].

Our study has some limitations. STEC was not isolated from all patients. Fecal STEC excretion in infected patients generally only lasts a few days [33], and our patients were admitted to the hospital after a median of 5 days of symptoms. STEC laboratory diagnostics have improved over time. During the early years of the study period, mainly culture-confirmed STEC cases were notified by clinical laboratories, whereas PCR-positive but culture negative cases were not before year 2013. This may underestimate the true number of STEC infections. On the other hand, since 2007, guidelines to control STEC infection have recommended screening of family members of patients with STEC infections. The retrospective nature of the study also means that in some cases, laboratory or clinical data are missing. Due to the small Finnish population (total 5.5 million), the number of HUS cases remains low.

To conclude, HUS is a rare but severe disease typically affecting young children. Over half of the patients need dialysis, the risk being greatest in children under 3 years of age. Besides young age, high leukocyte count is associated with the severity of the disease. *Stx2* and *stx2a* are linked to an increased risk of developing HUS, but the *stx* types do not predict the renal outcome or major CNS symptoms. Finally, approximately one quarter of the patients develop long-term renal or neurological sequelae.
